# Prevalences and predictive factors of maternal trauma through 18 months after premature birth: A longitudinal, observational and descriptive study

**DOI:** 10.1371/journal.pone.0246758

**Published:** 2021-02-24

**Authors:** Emilie Brunson, Aurore Thierry, Fabienne Ligier, Laurianne Vulliez-Coady, Alexandre Novo, Anne-Catherine Rolland, Julien Eutrope

**Affiliations:** 1 Université de Formation et de Recherche de médecine, Université de Reims Champagne-Ardenne, Reims, France; 2 Unité d’Aide Méthodologique, Hôpital Robert Debré, Centre Hospitalier Universitaire de Reims, Reims, France; 3 Pôle Universitaire de Psychiatrie de l’Enfant et de l’Adolescent, Centre Psychothérapique de Nancy, Laxou, France; 4 Unité de recherche EA 4360 APEMAC Adaptation, Mesure et Evaluation en Santé, Approches Interdisciplinaires, Université de Lorraine, Nancy, France; 5 Service de Psychiatrie de l’Enfant et de l’Adolescent, Hôpital Saint Jacques, Centre Hospitalier Universitaire de Besançon, Besançon, France; 6 Centre de Recherches Psychanalyse, Médecine et Société, Institut des Humanités, Sciences et Sociétés, Université de Paris, Paris, France; 7 Service de Psychothérapie de l’Enfant et de l’Adolescent, Hôpital Robert Debré, Centre Hospitalier Universitaire Reims, Reims, France; 8 Département de Psychologie, Laboratoire Cognition Santé Société (EA 6291), Université de Reims Champagne-Ardenne, Reims, France; University of Liverpool, UNITED KINGDOM

## Abstract

Posttraumatic reactions are common among mothers of preterm infants and can have a negative influence on their quality of life and lead to interactional difficulties with their baby. Given the possible trajectories of posttraumatic reactions, we hypothesized that prevalences of postpartum posttraumatic reactions at given times underestimate the real amount of mothers experiencing these symptoms within 18 months following delivery. Additionally, we examined whether sociodemographic and clinical characteristics of dyads influence the expression of posttraumatic symptoms among these mothers. A sample of 100 dyads was included in this longitudinal study led by 3 french university hospitals. Preterm infants born before 32 weeks of gestation and their mothers were followed-up over 18 months and attended 5 visits assessing the infants’ health conditions and the mothers’ psychological state with validated scales. Fifty dyads were retained through the 18 months of the study. The period prevalence of posttraumatic reactions was calculated and a group comparison was conducted to determine their predictive factors. Thirty-six percent of the mothers currently suffered from posttraumatic symptoms 18 months after their preterm delivery. The 18 months period prevalence was 60.4% among all the mothers who participated until the end of the follow-up. There was a statistical link between posttraumatic symptoms and a shorter gestational age at delivery, C-section, and the mother’s psychological state of mind at every assessment time. Only a small proportion of mothers were receiving psychological support at 18 months. Preterm mothers are a population at risk of developing a long-lasting postpartum posttraumatic disorder, therefore immediate and delayed systematic screenings for posttraumatic symptoms are strongly recommended to guide at-risk mothers towards appropriate psychological support.

## Introduction

### Background

Over the past decades, the frequency of premature live births has increased worldwide. A preterm birth is defined by the World Health Organization (WHO) as a delivery occurring before 37 completed weeks of gestation, including 3 subcategories: moderate or late preterm (32 to 37 weeks of pregnancy), very preterm (28 to 32 weeks) and extremely preterm (<28 weeks) [1]. In 2014, based on data from 107 countries, Chawanpaiboon et al. indicated an estimated worldwide number of 14.8 million preterm births, representing almost 10.6% of all live births, ranging from 8.7% in Europe to 13.4% in North Africa [2]. The same research team had previously estimated a 9.8% global rate in 2000. This increasing number of preterm live births may be related to the continuing medical progress prior to pregnancy including the development of assisted reproduction technologies (ART), during the pregnancy with obstetric care and also during the neonatal period thanks to neonatal intensive care. Several studies demonstrate that ART induced pregnancies are at higher risk of premature delivery, even in singleton pregnancies, compared to the general population [3–5].

With more viable births associated with preterm delivery, prematurity remains a major Public Health problem because of its negative impact on infant and childhood morbidity and mortality. In a systematic analysis in 2010, Liu and al. revealed that preterm birth and its complications were the second leading cause of child death, after pneumonia, among children under the age of 5 [6]. Infant mortality is negatively correlated to the term of the pregnancy [7, 8]: for example, in 2006 in the USA, mortality reached 2.4 per 1000 live births at 37–41 weeks of pregnancy, and it increased to 175.9 per 1000 live births under 32 weeks of gestation [9]. Morbidity among preterm children is significantly associated with immediate adverse health outcomes directly linked to the immaturity of the organ systems and intensive, frequently invasive interventions in the Neonatal Intensive Care Unit (NICU) for their survival. It is also associated with other long term sequelae such as developmental, psychological, emotional or behavioral problems leading to high healthcare costs [9–11].

Multiple studies indicate that aspects of premature delivery and the birth of a premature infant, such as hospitalization in a Neonatal Intensive Care Unit, the intensive and/or invasive care required by preterm infants, the separation of the mother-child dyad in combination with the mother’s fear for her child’s viability and his/her future are noteworthy sources of maternal psychological distress [12–15]. In a recent study, Schecter et al. questioned parents regarding specific stress factors in neonatology and the top three were: separation from the infant, the sight of intravenous or feeding tubes and the inability to hold their child [16]. Mothers of preterm newborns endure more severe levels of psychological distress than mothers of full-term babies [17, 18]. In 2013, Beck, Driscoll, and Watson defined traumatic childbirth as “an event occurring during the labor and delivery process that involves actual or threatened injury or death to the mother or her infant” during which “the birthing woman experiences intense fear, helplessness, loss of control and horror” [19]. Giving birth prematurely and having their newborn hospitalized in the NICU is a potentially traumatic event for mothers and may lead to the appearance of acute stress symptoms and even to posttraumatic stress disorder (PTSD) which is a mental disorder that can occur in people who have directly experienced or witnessed a traumatic event (or are close to a person who had such an experience) [20]. People suffering from PTSD endure intrusive and distressing re-experiencing of the trauma, negative changes in cognition and mood, avoidance of trauma-related reminders and arousal/reactivity symptoms [21]. Within the *Diagnostic and Statistical Manual of Mental Disorders 5* (DSM-V), PTSD has been relocated into a new diagnostic category “Trauma and Stressor-related Disorders” and is no longer among the anxiety disorders [21].

In 2014, a meta-analysis reported that the mean prevalence of postpartum PTSD among community samples was 3.1%, and was five times higher (15.7%) among at-risk samples without recording any specific prevalence attributed to preterm birth [22]. Another systematic review of the literature written by Gondwe and Holditch-Davis in 2015, revealed that the prevalence of elevated posttraumatic symptoms, and of a possible PTSD, ranged from 18% to 77.8% among mothers who delivered prematurely [23]. In 2017, Beck and Harrisson observed a similar range (from 14% to 79%) [24]. Our own previous researches revealed patterns consistent with previous literature [25, 26]. In fact, we observed that 40.2% of the mothers who delivered before 32 weeks gestational age experienced significant posttraumatic symptoms 6 months after delivery, and 34% at 12 months. We also demonstrated that posttraumatic symptom severity 6 months after discharge was correlated with the delivery conditions (C-section), the newborn’s clinical characteristics (Perinatal Risk Inventory [PRI], intrauterine growth restriction) and the anxio-depressive state of the mother just after the delivery, before discharge and 6 months later. Comparatively, the severity of posttraumatic symptoms at 12 months was also correlated with the delivery conditions (C-section) and positively correlated with the scores of anxiety and depression of the mother at all assessment times but was not correlated any longer with the characteristics of the preterm child at birth. The literature on the subject is quite contradictory: on one hand, many articles state maternal, infant or external factors as predictors of postpartum PTSD, such as anxiety or depression symptoms in mothers, young maternal age, higher maternal education, C-section, young gestational age, birth weight, length of stay at the hospital, less social support in the perinatal period [27–32]. On the other hand, some other authors found no significant association between postpartum PTSD and these factors [33, 34].

The mental health of mothers and in particular maternal posttraumatic stress, depression, and anxiety have been reported by several studies to have an impact on the quality of the mother-infant interactions and thus on the infant’s developmental and behavioral outcomes [35]. Our previous study revealed a positive correlation between the PIPE score (Pediatric Infant Parent Exam) at 12 months and the mental condition of the mother at 6 months (evaluated with the modified Perinatal Post-traumatic stress disorder Questionnaire [mPPQ] score, Hospital Anxiety and Depression Scale [HADS] scores and Edinburgh Post-natal Depression Scale [EPDS] scores), indicating less favorable interactions between the mother and her child if the mother experienced psychological distress during the postpartum period [26]. Other studies reveal different dyadic interactions between mothers and their child depending upon the term of the delivery: mothers of preterm infants with high posttraumatic symptoms are found to be more intrusive, more controlling and less sensitive in their interactions with their baby at 6 months of infant’s corrected age, while preterm babies tend to be more passive than full-term infants [36, 37]. Mothers of preterm infants suffering from posttraumatic symptoms also have more distorted representations of their infant than full-term mothers: they tend to view their child as less warm towards them, more invasive and more difficult in temperament [38–40]. Given these possible negative consequences for the mothers’ psychological states, the preterm infants’ development, and the dyads’ interactions: it is essential to detect every mother who is potentially experiencing posttraumatic symptoms and direct them to appropriate psychological support.

### Objectives

**Fig 1:**
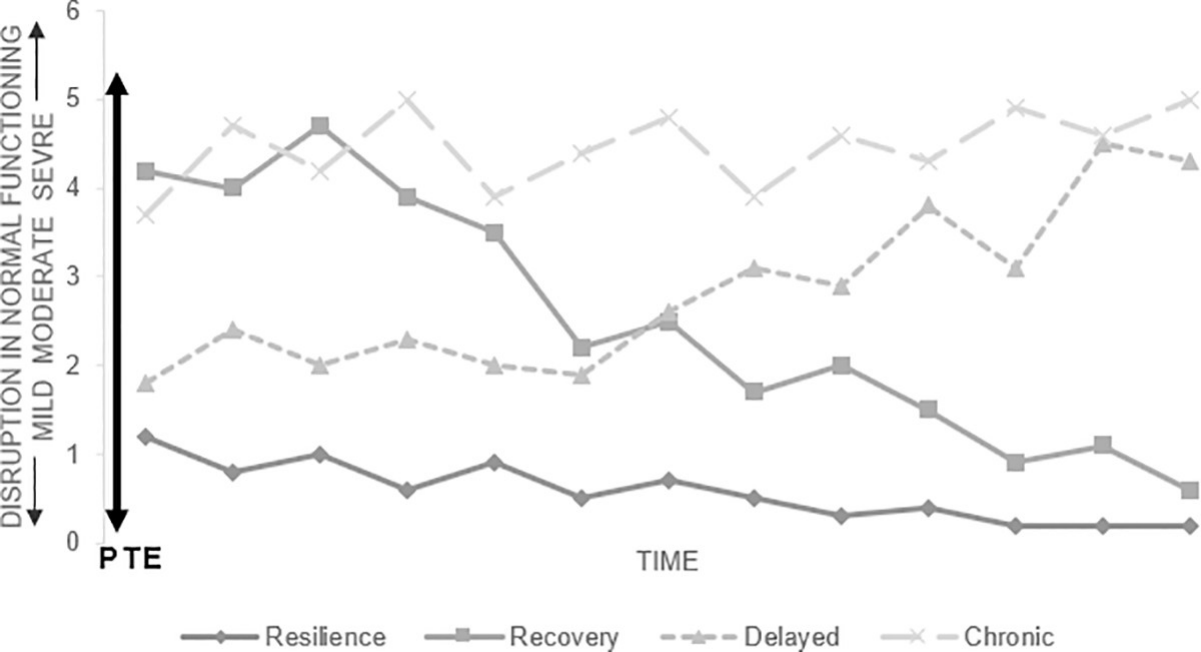
Possible outcome trajectories following an isolated potentially traumatic event. Based on Bonanno’s work in « Loss, trauma and human resilience: have we underestimated the human capacity to thrive after extremely aversive events? » [41]. PTE: potentially traumatic event.

Our present study is aimed to estimate the 18 months period prevalence of mothers affected by posttraumatic symptoms after they gave birth prematurely and search for predictive factors. Bonanno, a professor in Clinical Psychology at Colombia University who is studying how human beings cope with trauma identified, based on multiple longitudinal studies, 4 possible different outcome trajectories following an isolated potentially traumatic event (Fig 1): chronic dysfunction (elevated PSTD symptoms maintained over time), delayed PTSD (symptoms worsening over time), recovery (initial dysfunction but symptoms decreasing over time) and resilience (minimal disruption or absence of significative symptoms) [41]. In 2013, the DSM-V revised the PTSD diagnostic criteria: it removed the notion of acute and chronic PTSD and it replaced the concept of delayed-onset with “delayed expression” defined as “the full diagnostic criteria are not met until at least 6 months after the event (although the onset and expression of some symptoms may be immediate)” [21].

We assumed that in our study, mothers of preterms also follow these trajectories: the expression of the PTSD symptomatology might be delayed for some mothers while some who are initially suffering from significant posttraumatic symptoms might recover; thus, prevalence at a given time isn’t fully representative of how frequent it is and how many mothers actually suffer from postpartum posttraumatic symptoms within the few months following the preterm delivery. Therefore, our main hypothesis is that the 18 month period prevalence of significant posttraumatic symptoms in mothers following their preterm delivery is in fact higher than the point prevalences previously found at any given time of the study, because of the different possible posttraumatic psychological trajectories. Once we estimated this period prevalence, we want to determine if the expression of these symptoms is significatively linked to any of the socio-demographic and clinical characteristics of the dyads.

## Materials and methods

### Study design

This longitudinal prospective study was performed between January 2008 and January 2011 in 3 french university hospitals (Reims, Nancy, and Besançon) and is part of a larger research program. Its design has already been described more precisely in two previous articles [25, 26]: one hundred dyads made up of mothers who delivered prematurely before a 32 weeks gestational age and their preterm children were included in the study. The dyads were followed-up for 18 months and during this period attended 5 visits (Table 1): the first one in the maternity ward after the birth between day 1 and day 15, the second one in the neonatology ward within two weeks of the child’s discharge and then 3 other visits at 6, 12 and 18 months within the systematic tracking of premature newborns at the CAMSP (early medico-social action’s center). Every visit entailed an assessment of the mother, the child, and the dyadic interactions.

**Table 1:** Study design: 5 assessment times.

	Visit 1	Visit 2	Visit 3	Visit 4	Visit 5
Places	Maternity or neonatalogy	Neonatology	CAMSP		
Time	0 to 15 days after birth	Within 15 days before discharge	6 months	12 months	18 months
**Demographic data**	X	X	X	X	X
**mPPQ (mothers)**		X	X	X	X
**HADS (mothers)**	X	X	X	X	X
**PRI (newborn)**	X	X			

CAMSP: early medico-social action’s center

mPPQ: modified Perinatal Post-traumatic stress disorder Questionnaire, HADS: Hospital Anxiety and Depression Scale, PRI: Perinatal Risk Inventory.

Firstly, the point prevalence of mothers suffering from posttraumatic symptoms 18 months after they gave birth before term (visit 5) was calculated and statistical analyses were performed to determine if these traumatic symptoms at visit 5 were significantly associated with the dyads’ sociodemographic or clinical data.

Secondly, the 18 months prevalence of posttraumatic symptoms was calculated among mothers who participated and replied to the trauma questionnaire at every visit from visit 2 to visit 5. Participants were divided into 2 groups: 1. mothers who never had significant posttraumatic symptoms for the duration of the study and 2. mothers who had symptoms indicative of postpartum PTSD at least once during the study period. Between group statistics were performed to detect potential differences and identify predictive factors for postpartum posttraumatic stress reactions.

Mothers with acute or chronic psychological illness, drug or alcohol abuse, underage, and/or with difficulties understanding or speaking French were excluded. The exclusion criteria for the newborns included short term life-threatening conditions, malformation and/or genetic abnormalities diagnosed before the discharge from NICU, and/or vulnerability of the baby evaluated by Perinatal Risk Inventory *⩾* 10 [42], a score used to identify vulnerable children at risk of developing neurological developmental disorders.

This study was approved by the Institutional Review Board (IRB) of Reims University Hospital and written consents was collected after patients were provided with specific information regarding the study.

### Assessment

Sociodemographic and clinical data for both the mother and the newborn were collected at inclusion. The mother’s psychological condition and the mother-infant interactions were evaluated with multiple validated scales by a member of the research team as explained below.

Immediately after a premature birth, the child was initially examined by a pediatrician who evaluated them with the perinatal Risk Inventory (PRI) which is an 18 item scale developed by A. Scheiner et al. in 1991 predicting the risk of impaired outcomes in preterm newborns, based on perinatal factors such as gestational age, weight, cranial perimeter, Apgar scores (measures of the infant’s condition after birth, a score of 7 or more is normal), etc [42]. Each item is quoted from 0 to 3, a cut-off score of 10 or above indicates high perinatal risk, and higher scores suggest greater risks of developmental abnormality [42].

Mothers were assessed at each evaluation time from visit 2 to visit 5 with the modified Perinatal Posttraumatic stress disorder Questionnaire (mPPQ). The PPQ is is a tool developed by DeMier, Hynan and their colleagues in the late 90s [30], and modified later by Callahan et al. [43]. The French version has been translated by Pierrehumbert et al. in 2003 [44]. This questionnaire is used to evaluate the presence of posttraumatic symptoms in mothers of perinatal high-risk children, until 18 months after the delivery. The mPPQ consists of a 14 item self-report questionnaire, divided into 3 subgroups of questions rated from 0 (not at all) to 4 (often, for more than a month): questions 1 to 3 evaluate the presence of intrusion symptoms, questions 4 to 9 focus on active avoidance, loss of interest, isolation, and question 10 to 14 appraise hypervigilance, irritability and hypersensitivity. Questions are retrospective and the mothers answer regarding symptoms that appeared after the delivery. The total possible score on the mPPQ ranges from 0 to 56 and a cut-off of 19 or above identifies mothers with a high risk of a trauma experience. It is specific to the birth of a high risk child and can be expected to be more appropriate for the particular event of preterm birth.

Mothers’ psychological comorbidities such as anxious and depressive symptoms were also assessed at every visit. Therefore, the 2 subscales of the Hospital Anxiety and Depression Scales (HADS) developed by A. S. Zigmond and R. P. Snaith in 1982 [45] were also completed and a cut-off score of 8 or above was used to identify a clinically significant anxious and/or depressive symptomatology in mothers [46].

If scores were above cut-off levels for any of the previous scales, mothers were then addressed to psychiatrists, psychologists or psychomotor therapists for specific psychological treatment.

**Fig 2:**
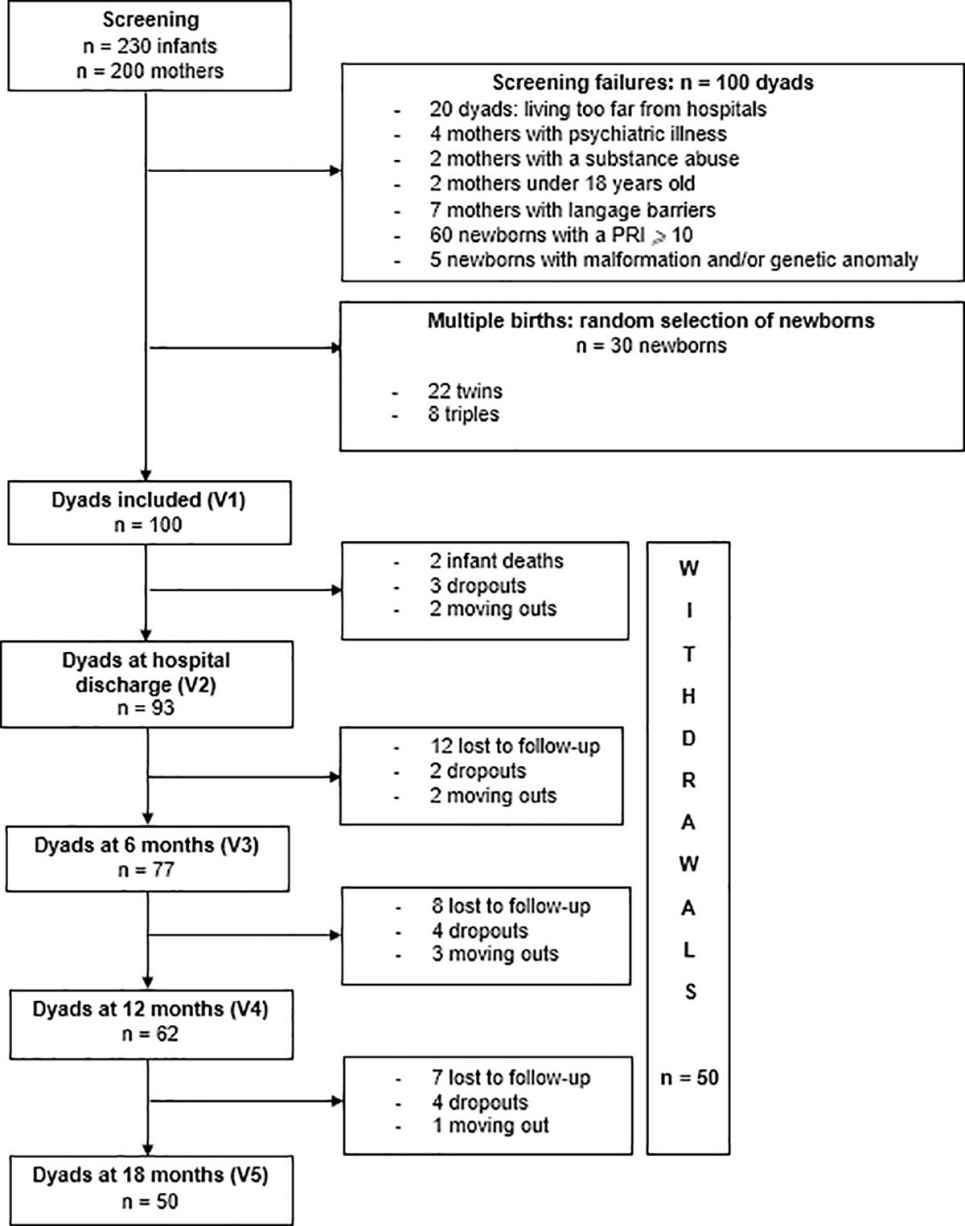
Flowchart of the study. PRI: Perinatal Risk Inventory; V1: assessment at inclusion; V2: assessment at the hospital discharge; V3: assessment 6 months after birth; V4: assessment 12 months after birth; V5: assessment 18 months after birth.

One hundred dyads, including mothers and their preterm child were included in the present study at visit number 1 immediately postpartum when they were still hospitalized in the maternity ward. The flowchart of the study illustrates the number of mothers assessed at each visit and the numbers of lost to follow-up, dropouts, moving outs, and infant deaths (Fig 2). At visit 5, 18 months after the preterm delivery, 50 dyads remained.

### Statistical analysis

Quantitative data were described as mean (m) and standard deviation (sd) while qualitative data were summarized by counts (n) and percentages (%). Univariate comparisons between the two groups were performed with Student t-test for quantitative variables. Khi^2^ test or Fisher test were used to evaluate statistical association between qualitative variables. A p-value of <0.05 was considered statistically significant. All statistical analyses were performed with SAS software version 9.4.

## Results

The final sample of 50 dyads at visit 5 was not significantly different from the initial population of 100 dyads at the inclusion (visit 1) in terms of mothers’ and infants’ characteristics (Table 2). The two groups were homogeneous. At visit 5, the mean age of the mothers when they gave birth was 30.9 ± 5.5 years, 40 (80%) had graduated from high school. It was the first pregnancy for 21 (42%) and 5 (10%) conceived through in vitro fertilization with their partner. Twelve mothers (24%) expected more than one baby and had a multiple pregnancy. Thirty-nine (78%) suffered from threatened preterm labor and 32 (64%) required C-section. The mean gestational age was 209.8 ± 10.9 days (30 weeks of gestation) and the mean birth weight was 1331 ± 350 grams. Twenty-five (50%) of the infants were males. Babies’ mean PRI score at birth was 4.9 ± 2.4 and 19 of them (38%) had an Apgar score below 7 at five minutes.

**Table 2:** Socio-demographic and clinical characteristics of the dyads at inclusion and at 18 months after delivery (during visit 5).

	Dyads at inclusion		Dyads at visit 5 (18 months)		p value
**Babys’ Characteristics :**					
Gestational age in days (m, sd)	n = 99	208.9 ± 11.6	n = 50	209.8 ± 10.9	0.65
Birth weight in grams (m, sd)	n = 100	1320 ± 330.6	n = 50	1331 ± 350	0.85
Sex (nb of males, %)	n = 100	51 (51%)	n = 50	25 (50%)	0.91
Apgar score < 7 at 5 min (nb, %)	n = 100	37 (37%)	n = 50	19 (38%)	0.90
Neonatal unit admission (nb, %)	n = 100	100 (100%)	n = 50	50 (100%)	1
PRI score at birth (m, sd)	n = 95	4.7 ± 2.5	n = 49	4.9 ± 2.4	0.65
**Mothers’ Characteristics :**					
Mother’s age in years (m, sd)	n = 100	29.8 ± 6	n = 50	30.9 ± 5.4	0.28
Graduated from high school (nb, %)	n = 97	74 (76,3%)	n = 50	40 (80%)	0.42
Primiparous (nb, %)	n = 100	48 (48%)	n = 50	21 (42%)	0.49
In vitro Fertilisation (nb, %)	n = 100	10 (10%)	n = 50	5 (10%)	1.00
Multiple pregnancy (nb, %)	n = 100	22 (22%)	n = 50	12 (24%)	0.78
Threatened preterm labor (nb, %)	n = 100	76 (76%)	n = 50	39 (78%)	0.78
Ceasarean section (nb, %)	n = 100	54 (54%)	n = 50	32 (64%)	0.24
HADS anxiety at V1 (m, sd)	n = 98	10.2 ± 4.4	n = 50	9.9 ± 4.2	0.69
HADS depression at V1 (m, sd)	n = 98	6.8 ± 4.3	n = 50	6.8 ± 4.4	1.00
HADS global at V1 (m, sd)	n = 98	17 ± 7.9	n = 50	16.6 ± 7.7	0.77

m: mean, sd: standard deviation, nb: number, %: percentage

PRI: Perinatal Risk Inventory, HADS: Hospital Anxiety and Depression Scale

At visit 5, every mother completed multiple questionnaires assessing their psychological distress (Table 3). The mean mPPQ score was 14.7 (SD = 10.3). Among the 50 mothers, 18 (36%) had an mPPQ score equal to or above 19. The mean HADS depression score was 4.1 (SD = 3.7) and 10 mothers (20%) had a HADS depression score above the cut off (8). The mean HADS anxiety score was 7 (SD = 3.5); 16 mothers (32%) had a HADS anxiety score above the cut off (8).

**Table 3:** Psychological assessment results for mothers 18 months after birth (during visit 5).

	Total nb of mothers	m, sd	Score above cut off (nb, %)
HADS anxiety	50	7 ± 3.5	16 (32%)
HADS depression (m, sd)	50	4.1 ± 3.7	10 (20%)
mPPQ (m, sd)	50	14.7 ± 10.3	18 (36%)

HADS: Hospital Anxiety and Depression Scale; mPPQ: modified Perinatal Post-traumatic stress disorder Questionnaire

m: mean, sd: standard deviation, nb: number, %: percentage

The mPPQ score at V5 was significantly associated with the psychological state of mind of the mother at V5 (p = 0.02 for the HADS depression; p = 0.04 for the HADS anxiety) (Table 4). There was no significant relationship between mothers’ mPPQ at visit 5 and the characteristics of the pregnancy or with the delivery mode: that is to say no significant relationship between C-section and posttraumatic reaction at visit 5 (p = 0.77).

**Table 4:** Association between mPPQ scores at V5 and characteristics of the dyads.

Primiparous	p = 0.79
In vitro Fertilisation	p = 0.64
Multiple pregnancy	p = 0.46
Threatened preterm labor	p = 0.49
C-section	p = 0.77
Psychological support at V5	p = 0.67
Graduated from high school	p = 0.46
HADS depression at V5	**p = 0.02**
HADS anxiety at V5	**p = 0.04**

V5: Visit 5

HADS: Hospital Anxiety and Depression Scale

**Fig 3:**
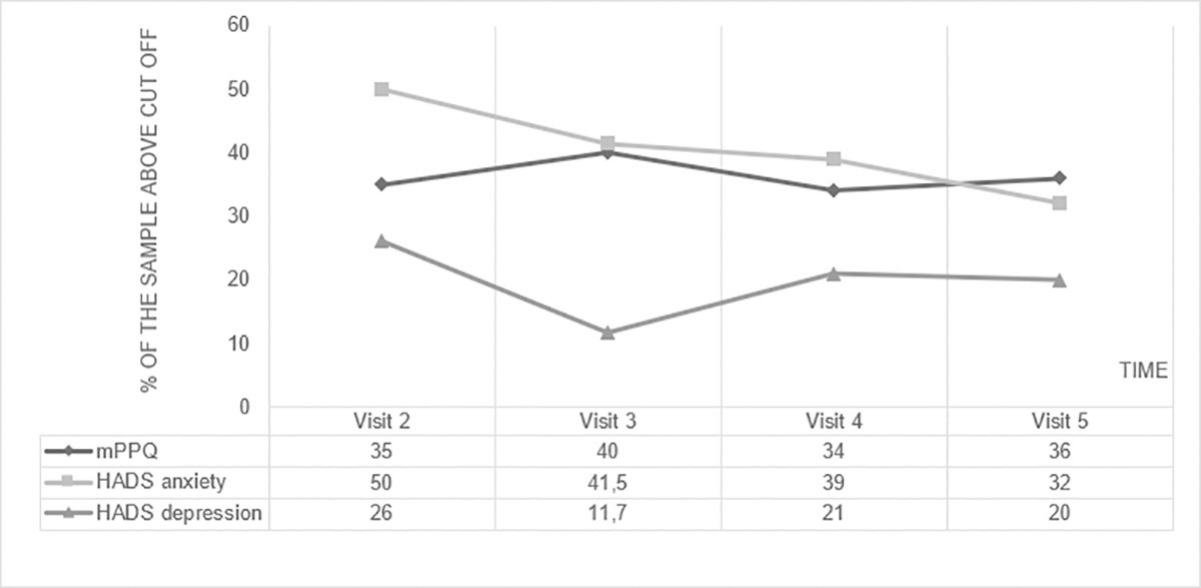
Point prevalences of mothers with significant posttraumatic symptoms (mPPQ score ≥ 19), anxious symptoms (HADS anxiety ≥ 8) and depressive symptoms (HADS depression ≥ 8).

During our study, the point prevalence of mothers affected by significant posttraumatic symptoms at every visit remained stable: 35% within two weeks before discharge, 40% 6 months after birth, 34% 12 months after birth, and still 36% at 18 months after birth (Fig 3). These results refer to prevalences at 4 different given times of the study.

To obtain the period prevalence (for the 18 months) as explained previously in the methods, the dyads were divided into 2 groups. Forty-eight mothers attended every visits from visit 2 to visit 5: the first group included 19 mothers (39.6%) with an mPPQ score below the cut off score at every visit and the second group included 29 mothers (60.4%) with an mPPQ at least once above the threshold. Socio-demographic and clinical characteristics of both groups can be found in Table 5.

**Table 5:** Comparisons of socio-demographic and clinical characteristics between mothers with no significant posttraumatic symptoms and mothers with any significant posttraumatic symptoms from V2 to V5.

	Mothers with mPPQ < 19 at every visit		Mothers with mPPQ ⩾ 19 at least once from V2 to V5		p value
	n = 19	39.6%	n = 29	60.4%	
**Babys’ Characteristics :**					
Gestational age in days (m, sd)	n = 19	213.8 ± 9.6	n = 29	207.4 ± 11.3	**0.0479**
Birth weight in grams (m, sd)	n = 19	1442 ± 325	n = 29	1250 ± 360	0.07
Sex (nb of males, %)	n = 19	9 (47,4%)	n = 29	15 (51,7%)	0.77
Apgar score < 7 at 5min (nb, %)	n = 19	7 (36,8%)	n = 29	12 (41,4%)	0.75
Neonatal unit admission (nb, %)	n = 19	19 (100%)	n = 29	29 (100%)	1
PRI score at birth (m, sd)	n = 18	4.4 ± 2.7	n = 29	5.2 ± 2.3	0.28
**Mothers’ Chartacteristics :**					
Mother’s age in years (m, sd)	n = 19	29.5 ± 3.8	n = 29	32 ± 6.2	0.12
Graduated from high school (nb, %)	n = 19	15 (79%)	n = 29	24 (82.8%)	1.00
Primiparous (nb, %)	n = 19	7 (36.8%)	n = 29	13 (44.8%)	0.58
In vitro Fertilisation (nb, %)	n = 19	3 (15.8%)	n = 29	2 (6.9%)	0.37
Multiple pregnancy (nb, %)	n = 19	5 (26.3)%)	n = 29	5 (17.2%)	0.49
Threatened preterm labor (nb, %)	n = 19	13 (68.4%)	n = 29	24 (82.8%)	0.30
Ceasarean section (nb, %)	n = 19	9 (47.4%)	n = 29	22 (75.9%)	**0.0435**
HADS anxiety score ⩾ cut off at V2 (nb, %) at V3 (nb, %) at V4 (nb, %) at V5 (nb, %)	n = 18 n = 19 n = 19 n = 19	7 (38.9%) 5 (26.3%) 1 (5.3%) 3 (15.8%)	n = 26 n = 29 n = 29 n = 29	21 (75%) 16 (55.2%) 17 (58.6%) 13 (44.8%)	**0.0143** **0.0487** **< 0.001** **0.037**
HADS depression score ⩾ cut off at V2 (nb, %) at V3 (nb, %) at V4 (nb, %) at V5 (nb, %)	n = 18 n = 19 n = 19 n = 19	0 (0%) 0 (0%) 0 (0%) 2 (10.5%)	n = 28 n = 29 n = 29 n = 29	14 (50%) 7 (34.1%) 12 (41.4%) 8 (27.6%)	**< 0.001** **0.0326** **0.0013** 0.28
Psychological support at V2 (nb, %) at V3 (nb, %) at V4 (nb, %) at V5 (nb, %)	n = 19 n = 15 n = 16 n = 12	3 (15.8%) 2 (13.3%) 2 (12.5%) 2 (16.7%)	n = 29 n = 23 n = 21 n = 18	12 (41.38%) 4 (17.4%) 4 (19%) 5 (27.8%)	0.06 1.00 0.68 0.67

V2: visit 2, V3: visit 3, V4: visit 4, V5: visit 5

HADS: Hospital Anxiety and Depression Scale, mPPQ: modified Perinatal Post-traumatic stress disorder Questionnaire

There was no significant differences between groups based on mothers’ age at birth, educational level, characteristics of the baby at birth (sex, weight, sex, Apgar scores, PRI score at birth), nor were there any significant differences between groups in terms of parity, in vitro fertilization, multiple pregnancies and threatened preterm labor. Further, there were no between-group differences in the proportions of mothers who received a psychological support.

Mothers with posttraumatic symptoms delivered at a significatively earlier gestational age (p = 0.0479): indeed, they delivered at a mean of 207.4 ± 11.3 days (29.6 weeks of gestation) while those with no significant posttraumatic symptoms delivered at a mean of 213.8 ± 9.6 days (30.5 weeks of gestation). The delivery mode was also significatively different between both groups (p = 0.0435): there were more C-sections among the group of mothers who experienced posttraumatic symptoms during the study. Seventy-five percent required C-section compared to 47.4% in the group with no significant traumatic symptoms. Mothers with posttraumatic symptoms displayed higher HADS anxiety scores at every visit and higher HADS depression scores until visit 4 but this trend was not observed at visit 5.

## Discussion

### A persistent traumatic impact

Our results indicate that 36% of the mothers who delivered before 32 weeks of gestation had an mPPQ score above the threshold indicating that they were currently suffering from significant posttraumatic symptoms 18 months after their preterm delivery. PTSD is a disorder that can affect people for years after the initial trauma [47]: here, the prevalence of mothers with a traumatic reaction at 18 months remains high and shows that premature birth is a potentially traumatic event that can lead to a long lasting psychological distress in mothers. In addition, 32% of mothers experienced anxious symptoms and 20% depressed symptoms, evaluated with the HADS subscales 18 months after birth. Mothers with a traumatic reaction at 18 months also experienced more clinically significant anxious and depressive symptoms than mothers without a traumatic reaction. This significant relation was previously observed in our earlier article on traumatic reactions at 6 and 12 months post-partum [26]. While the traumatic reaction was significantly linked to the delivery mode until 12 months after delivery, and with the presence of an intrauterine growth restriction at 6 months [26]: we didn’t observe any significant link between the presence of significant posttraumatic symptoms at 18 months and any of the infant’s clinical characteristics or mothers’ sociodemographic characteristics, or with their medical history during pregnancy.

Only a few studies closely examined the evolution of posttraumatic symptoms in mothers over time, and those limited results are contradictory: some suggest that posttraumatic symptoms prevalences are stable [20] while others indicate that prevalences decrease over time [48]. In our present study, for the 18 months of the follow-up, we observed stable point prevalences of high posttraumatic symptoms among mothers of preterm infants; from 35% within 2 weeks before discharge to 36% at 18 months post-delivery. Considering Bonanno’s work [41] describing the different trajectories of possible posttraumatic psychological reactions after a potentially traumatic event (Fig 1), our assumption was that mothers of preterm infants would also follow these trajectories, with some mothers recovering from initial posttraumatic symptoms and other with delayed expression of posttraumatic symptoms. A cross-sectional look at a point prevalence is then not representative of every mother who experience or is at risk of experiencing posttraumatic symptoms during the 18 months postpartum period but may be only the tip of the iceberg. Our hypothesis was supported given that we demonstrated that during the 18 months follow-up, 60.4% of our study population experienced significant posttraumatic symptoms related to their traumatic delivery, revealing that posttraumatic reactions in mothers who give birth to preterm infants are numerous and should be of great concern in healthcare.

### Predictive factors

Among the mothers (60.4%) suffering from posttraumatic symptoms we found a significant link between these symptoms and the delivery characteristics (C-section), the baby’s characteristics (shorter gestational age) and the mothers’ psychological state of mind at every assessment time. Multiple studies, including our previous article [26] demonstrate that C-section is a meaningful risk factor for postpartum PTSD and our findings here concur [49]. Our findings also suggest that the earlier the gestational age is at the time of delivery, the greater the risk of the development of maternal posttraumatic symptoms. In fact, at this point of their pregnancy, most women are not actively anticipating immediate delivery and might feel unprepared to face this unexpected event. A shorter gestational age implies a smaller chance of survival for the baby, eliciting significant fear from mothers for their infant’s viability. Since the first criteria for PTSD is experiencing an actual or threatened injury to oneself or a relative [21], we can assume that C-section, and even more so, an emergency C-section, as well as a shorter gestational age along with uncertainty regarding their infant’s viability, may lead to the development of a PTSD in mothers of preterm infants. Our current findings also revealed that mothers experiencing a traumatic reaction were significantly more anxious before NICU’s discharge, at 6, 12 and 18 months and they were also significantly more depressed before NICU’s discharge, at 6 and 12 months but not at 18 months. Additionnaly, only a small proportion of mothers suffering from posttraumatic symptoms receive psychological support (27.8%), which indicates there is either an issue with evaluation and orientation by the medicosocial team or a lack of compliance with such service by the mothers.

### Clinical implication

Given that PTSD is a chronic disorder, and since the expression of its symptoms can be delayed months or even years after the potentially traumatic event, it is essential that every mother who delivers prematurely undergoes early psychological screening during which posttraumatic symptoms could be detected. For many years, immediate psychological debriefing support has been routinely offered to victims of potentially traumatic events but recent studies indicate that it may be ineffective or even detrimental. A Cochrane Review from 2002 found no evidence that debriefing reduces the risk of the development of PTSD, and trials with the longest follow-up suggest that it could even result in adverse effects [50]. Many specific intervention programs have been developed especially for parents of preterm children and demonstrate their efficacy in preventing, or helping reduce, psychological distress in parents and developmental problems in infants [51–56]. A meta-analysis by Benzies et al. suggests that an early effective intervention after preterm birth was based on three key components: parents’ support, parents’ education, and child’s development support [57]. These programs have been proven to shorten the hospital stay in NICU and reduce health care costs.

In multiparous mothers, a history of a previous preterm delivery increases the risk of another preterm delivery by 2 to 5 times [58]. Considering that a history of prior exposure to trauma is a significant risk factor for PTSD, mothers who previously experienced a preterm delivery are at increased risk of experiencing another preterm delivery and thus, have a higher risk of developing substantial posttraumatic symptoms [59–61]. These mothers with a history of preterm deliveries should, therefore, be targeted for psychological screenings, given that they are a population at higher risk.

Further research should address the optimal time for assessments of at-risk mothers, since there are multiple potential trajectories of psychological reactions after trauma exposure, with an immediate or delayed expression of distress. It is essential to offer medical and psychological support for mothers who express immediate significant traumatic symptoms that can be screened for in the neonatology ward, to prevent the chronicization of posttraumatic symptoms which might interfere with their normal functioning, degrade their quality of life and impact the mother-infant relationship and the child development. For the mothers who are not initially flagged at risk by the early screening due to insufficient symptoms, follow-up screenings should be systematically implemented. For example, screenings of mothers could easily be implemented within the systematic tracking of premature newborns at the CAMSP at 6, 12 and 18 months so they can be referred to a psychological clinician or a psychiatrist.

### Strengths and limitations

The strengths of our study are the large sample, the length of the follow-up of this at-risk population of preterm dyads which allowed us to assess mothers 5 times over an extended period, and the validated scales used to evaluate the dyads. We also identified a few limitations in our study. First, the number of participants of lost-to follow up reached half of the included dyads over the 18 months of the study (due to infants death, moving-outs, drop-outs). Second, even if mothers with acute or chronic psychological illnesses were excluded at inclusion, the medical interview took place after the delivery, and thus the prior anxious and depressive states of the mother remain uncertain. It would have been interesting had we been able to screen mothers before the delivery, however, this was not possible given preterm deliveries are often unexpected and urgently addressed to save the mother and her unborn child.

## Conclusion

Posttraumatic symptoms occurring after a preterm delivery are frequent and recorded within 60.4% of our study population during the 18 months postpartum follow-up. Posttraumatic reactions are significantly linked to C-section, earlier gestational age at the time of delivery, and the mother’s anxious and depressive symptoms. Given the potential traumatic reaction’s trajectories, immediate and delayed systematic screening should be implemented. Further researches should address the optimal timeframe for these follow-up screenings of at-risk mothers in order to guide them towards appropriate psychological support.
